# Mental health problems and suicidal expressions among young male prisoners in Cambodia: a cross-sectional study

**DOI:** 10.1080/16549716.2021.1985229

**Published:** 2021-10-13

**Authors:** Puthy Pat, Linda Richter-Sundberg, Bhoomikumar Jegannathan, Kerstin Edin, Miguel San Sebastian

**Affiliations:** a Center for Child and Adolescent Mental Health (Caritas-CCAMH), Cambodia; b Department of Epidemiology and Global Health, Umeå University, Umeå, Sweden

**Keywords:** Mental health problems, suicide expressions, young prisoners, Cambodia

## Abstract

**Background:**

incarceration and mental health problems are known to have a strong empirical association. Many studies have confirmed the high prevalence of mental health problems among young prisoners in particular, yet none has been conducted in Cambodia.

**Objectives:**

this study aimed to assess the level of mental health problems and suicidal expressions, and determine the associated risk factors among young prisoners in Cambodia.

**Method:**

this was a cross-sectional study among 572 young prisoners between the ages of 15 and 24 from three prisons. Sociodemographic data and detailed information on participants’ profiles were gathered, and mental health problems and suicidal expressions were assessed using the Youth Self-Report (YSR) and the Attitude Towards Suicide (ATTS) questionnaires, respectively.

**Results:**

Mental health problems as revealed by the mean YSR scores were: 25.97 for internalizing and 18.12 for externalizing problems; 11.88 for anxiety/depression, 9.97 for aggressive behaviours and 7.53 for somatic complaints. Social problems, attention problems and rule breaking behaviour were in the range of 8.10 to 8.49. Withdrawal depression and thought problems mean scores were 6.55 and 6.66, respectively. Mental health problems were associated with younger age, lower educational background, and shorter duration of incarceration. Around 16% had thought about their own death, and 12% expressed wish to die. Suicide ideation, planning, and attempts were reported by almost 7%, 2%, and 3% of participants respectively. Prior drugs users thought about death significantly more than their counterparts while suicide ideation was significantly lower among prisoners with higher education.

**Conclusion:**

Mental health problems and suicidal expressions among young prisoners warrant well-planned mental health services that are integrated into the current prison health system. A contextualised intervention that takes into account age, education, duration of incarceration and previous drug use may contribute to improve the mental well-being of young prisoners in Cambodia.

## Background

The prevalence of mental disorders in prisons is much higher than in the general population and there is a strong association between being incarcerated and mental health problems [1]. Studies from Europe and Africa have shown significant levels of mental health problems among prisoners [2–4]. Similarly, in Asia, studies from Hong Kong and India have reported a high prevalence of mental health problems among prisoners compared to the general population [5,6]. A systematic review of the prevalence of mental health problems in prisons from 13 different low and middle-income countries (LMICs) reported 15.8 times higher rates of non-affective psychosis among prisoners than in the general population [7]. Another systematic review and meta-analysis of published studies from 27 countries that included 35,361 suicides highlighted the importance of institutional factors associated with suicides [8]. Having no social status, serving life sentence and types of offence, in particular homicide, were the strongest factors associated with current suicide ideations [8]. A study from Australia revealed that the prevalence of suicidal expressions among the incarcerated was much higher compared to the general population with one-third of the prisoners reporting suicidal ideation and one-fifth reporting suicide attempts [9].

Reasons for the high prevalence of mental disorders among prisoners are multifaceted; overcrowded prison settings constitute one important cause but others may include loss of autonomy, decision-making, and freedom [10,11]. Moreover, pre-incarceration factors such as poverty, homelessness, unemployment, lack of education, substance abuse, and previous mental illness are all factors that partly explain the high prevalence of psychological problems among this population [10]. It is also known that age is an important factor to understand mental health issues, and young prisoners are particularly vulnerable to mental health problems, having higher prevalence rates than older age groups [12].

Most of the studies in prison settings, particularly among young people, are from high-income regions such as North America and Europe. A study among the first-time juvenile offenders in the USA revealed high rates of mental disorders, as nearly three quarters met the criteria for a mood, anxiety or behaviour disorder [13]. Hofvander et al [14] reported that almost 93% of the respondents between the age of 18 to 25 years in Swedish prisons met the criteria for at least one Axis I disorder (emotional and behavioural disorders), among which mood (54%) and anxiety (52%) disorders were the most common mental health problems. Another study on mental health problems among male young offenders in custodial versus community based-programs from Portugal reported that 91.2% of their respondents fulfilled the criteria for at least one psychiatric disorder [15]. Only a few studies have been conducted in the Asian context. A study among juvenile offenders in 23 correctional institutions from Taiwan reported that 44.1% of them had psychological problems such as sleep disturbance (36.8%), depression (34.7%), and hostility (27.9%) [16]. Zhou et al [17] investigating the mental health problems among juvenile boys aged 15–17 years in China found that 81.0% met the criteria for mental disorder including disruptive behaviour disorder (80.2%) and substance use disorder (SUD) (22.4%) with high levels of comorbidity (38.8%). The study among Malaysian juvenile offenders reported that 93.3% of the respondents had at least one diagnosable mental health condition and 76.2% had two or more psychiatric diagnoses, in which conduct disorder was the most common mental disorder (59.0%), while SUD the most common co-morbidity [18].

Mental health problems among young prisoners have a significant impact on their social, economic, educational, vocational, interpersonal and physical status lifelong [11,19]. Research has shown that antisocial behaviours often continue with increased risk of reoffending and negatively affecting parenting skills, even across generations after release from prison [19,20]. A study assessing the needs of young prisoners in England and Wales found that 79% of them needed mental health and social relationship supports, while only 36% for educational or work needs. Therefore, a structured needs assessment process while in the prison settings is important to address the mental health needs of young prisoners to improve their quality of life [21].

Suicidal behaviours among young prisoners are reported, though not as widely as among adults. A global scoping review by Borschmann et al [22] that looked into 245 publications listed the following lifetime prevalence of health problems among adolescents in detention: neurodevelopmental disorders (2–47%), infectious diseases (0–34%), sexual and reproductive problems (20–37%), mental health (0–95%) and substance abuse problems (22–96%), and self-harm (12–65%). A longitudinal study among 1,829 young people from the age of 10 to 18 in a juvenile temporary detention centre in the US reported one in ten juvenile detainees thought about suicide and 11% attempted. This study also reported that incarcerated youths died by suicide two to three times more than young people in the general population [23]. Another study from Australia among young prisoners observed that 60% of them reported suicide thoughts and 5% attempted suicide in the past year while 10% reported suicide attempts in their lifetime [24]. Though there are some studies on mental health of young prisoners from LMICS, few are on suicidal expressions. A study from Pakistan among young prisoners reported that 22% of them had suicidal thoughts [25].

It is crucial to identify and assess the needs and types of mental health problems in young prisoners soon after imprisonment in order to provide effective and timely mental health services for them [26]. However, the majority of the prison mental health research has been conducted in high-income countries and among adults [27,28]. To our knowledge, the literature on mental health problems, suicidal expressions and related risk factors among young prisoners in LMICs is scarce, particularly in post-conflict settings. This study aimed to assess the mental health problems and suicidal expressions and determine the risk factors associated with these two outcomes among young prisoners in Cambodia, a country located in Southeast Asia with a history of more than 30 years of civil unrest.

## Method

### Study setting and design

Cambodia is a post-conflict and low-income country with more than three decades of internal conflict. During the genocidal regime, the health system was destroyed and around two out of seven million died due to execution, torture, landmines, overwork, or starvation [29]. The health system in Cambodia has been revitalised gradually since 1979 [30]; however, it continues to remain underdeveloped [31], particularly in the prison setting. There are 24 prisons spread throughout the country, one in each province, with a total of 5,552 young prisoners between the ages of 15 and 24 [32]. This is a cross-sectional study among young prisoners conducted from January 2018 until August 2019 in Cambodia.

### Sampling

The three prisons, randomly selected from the total of 24 prisons, were located in Battambong, Kampong Cham and Kandal provinces. Battambong is in the northwest part of the country with a population of around one million. Kampong Cham is in the central lowlands of the Mekong river with a population of around 900,000. Kandal Province is in the southeast and an economic belt of the capital city, Phnom Penh, with a population of 1.2 million. All the provinces are predominantly rural with a similar sociodemographic profile, sustenance agriculture being the main occupation [33]. The prison data showed the number of female prisoners was less than 1% [32] and therefore we decided to recruit only young men in this study. All incarcerated young male prisoners who were on appeal or convicted and between the age group of 15 to 24 were invited to participate. Young prisoners under trial were excluded. The prison authorities provided a list of 739 prisoners who fulfilled the criteria and all of them were included in the study. However, 167 young prisoners were released during the interview period, making a total of 572 respondents. Among these, 100 were from Kampong Cham, 167 from Battambong and 305 from Kandal prisons. The participation was entirely voluntary and informed consent was obtained and confidentiality of the data was assured. The necessary ethical clearance was obtained as per the norms and regulation of conducting research in the prison setting in Cambodia (N33NGCHR).

### Measures

Sociodemographic information such as age, marital status, education, employment, and religion were collected. We used the Youth Self Report (YSR) and the Attitude Towards Suicide (ATTS) questionnaires to determine the mental health problems and suicidal expressions, respectively. The sociodemographic variables and other risk factors were recorded as follows: age in two groups: 15 to 19 and 20 to 24 years; marital status was noted as single, married, and separated or divorced; education was grouped into low (never attended school at all or only at primary school level), and high (7th grade and above); employment status prior to imprisonment was categorised as being employed (paid job) or unemployed (no paid job); and religion was categorized into Buddhist or others. We classified criminality as acquisitive crime (burglary and robbery), sexual abuse, drug-related, violent crime, and others. In cases where the young prisoners were charged with more than one crime, they were asked to select only the main one. Previous conviction referred to whether they had been imprisoned in the past or not. Time spent in the prisons so far and time left to serve were divided into two categories: less than one year and more than one year. History of alcohol consumption and drug use were dichotomised as ‘no’ if they never or rarely (less than once per month) drank alcohol or used drugs, and ‘yes’ if they drank alcohol or used drugs more than once per month.

YSR, a part of Achenbach system of empirically based assessment (ASEBA), captures a range of mental health problems such as anxiety-depression, withdrawal depression, somatic complaints, thought problems, social problems, rule-breaking behaviour, aggression, and attention problems. The above syndromes are coalesced to form internalising (anxiety-depression, withdrawal depression and somatic complaints) and externalising problems (rule-breaking and aggressive behavior) [34,35]. It consists of 112 items that were rated as 0 = not true, 1 = somewhat true, and 2 = often true. YSR has been used in previous studies among young people in different countries [36,37] including Cambodia. The cut-off score of YSR has not been validated in Cambodia and therefore, the mean score was considered for analysis as in previous studies [38,39].

ATTS has three parts: (1) exposure to suicidal behaviour from significant others such as relatives and/or friends, (2) attitude, belief, and misconceptions of suicide, and (3) one’s own suicidal expressions during the past one year which is the main focus of our study [40,41]. ATTS has been used in previous studies in other LMICs including Cambodia and we followed the scoring method as follows: 0 = never, 1 = hardly ever, 2 = sometimes and 3 = often. The scores were dichotomized into ‘No’ for never and hardly ever, and ‘Yes’ for sometimes and often as done in previous studies [37,38].

### Data collection process

Conventionally both YSR and ATTS are self-administered. We found that most of the prisoners had minimal education or were preliterate and therefore their ability to understand and respond to the questionnaires could be limited. In this context, the research team interviewed the participants instead of self-administering the questionnaires. Seven psychology students who had more than two years of experience in data collection in mental health research were recruited to conduct the structured interviews. Three days of face-to-face training on the application of the questionnaires were carried out that included role-play and use of illustrations while applying the questionnaires to bring uniformity in interview techniques and to minimise the interrater differences in administering the questionnaires. The interviews were conducted in specifically designated places in the respective prisons to provide a sense of privacy and confidentiality. The interview took approximately 30 to 40 minutes for each individual.

### Analysis

We applied descriptive statistics to calculate the means and frequencies of all variables. For the outcomes, the mean score of YSR and the prevalence of ATTS were estimated. To analyse the association between risk factors and both outcomes, regression analyses were applied in two stages. First, crude regression analysis was performed to determine the association between each of the independent variables and the mental health and suicide measures (YSR and ATTS). Linear regression was used for YSR and logistic regression for the ATTS outcomes. Statistically significant variables in these models were included in a multivariable regression analysis. The level of statistical significance was assessed using a 95% confident interval. We used Stata version 15 to analyse the data.

## Results

### Participants´ information

Table 1 shows the sociodemographic information of the participants. The majority of the young prisoners (60.84%) were between the age of 20 and 24. Most of them were single (84.62%) and 54.37% had completed secondary school and university graduation, noted as ‘high education’ in our study. Approximately two thirds were employed (75.94%) before they were incarcerated. The majority were Buddhist (92.66%). Drug-related crime (55.07%) was the most common reason for incarceration, followed by acquisitive (20.98%) and violent crimes (11.36%). Nearly two thirds (69.41%) of the young prisoners have been in the prison for less than one year and 60.49% reported the remaining period of incarceration to be less than one year. Most of the young prisoners (91.96%) reported to be in the prisons for the first time and had no previous conviction. Drug use and alcohol consumption before imprisonment were reported by 76.57% and 68.18% of participants, respectively.

**Table 1.** Profile of the participating young prisoners, Cambodia

Description	Frequency (N)	Percentage (%)
**Age**		
15 to 19	224	39.16
20 to 24	348	60.84
**Marital status**		
Single	484	84.62
Married	62	10.84
Separated/Divorced	26	4.55
**Education**		
Low education (no school & primary school)	261	45.63
High education (secondary school & university graduation)	311	54.37
**Employment status**		
Yes	424	74.13
No	148	25.87
**Religion**		
Buddhist	530	92.66
Other	42	7.34
**Types of criminalities**		
Drug related	315	55.07
Acquisitive crime	120	20.98
Violent crime	65	11.36
Sexual abuse	45	7.87
Others	27	4.72
**Period spent in prison so far**		
Less than one year	397	69.41
More than one year	175	30.59
**Duration continued to stay in prison**		
Less than one year	346	60.49
More than one years	226	39.51
**Previous conviction**		
No	526	91.96
Yes	46	8.04
**Drug use before imprisonment**		
No	134	23.43
Yes	438	76.57
**Alcohol consumption before imprisonment**		
No	182	31.82
Yes	390	68.18

### Mental health problems

Table 2 shows the mental health problems as measured by the YSR questionnaire. The mean for anxiety/depression was 11.88, 9.97 for aggressive behaviors, and social problems, attention problems and rule breaking behavior were in the range of 8.10 to 8.49. The somatic complaints mean score was 7.53 while withdrawal depression and thought problems were 6.55 and 6.66, respectively. Internalizing and externalizing problems mean scores were 25.97 and 18.12, respectively, and the total YSR mean score was 67.34.

**Table 2.** Mean YSR syndrome (SD) among young male prisoners, Cambodia

	Total (N = 572)
	Mean scores (SD)
Total YSR scores	67.34 (28.40)
Anxiety/depression	11.88 (5.11)
Withdrawal depression	6.55 (3.25)
Somatic complaints	7.53 (4.70)
Social problems	8.49 (4.41)
Thought problems	6.66 (4.33)
Attention problems	8.10 (3.76)
Rule breaking behavior	8.15 (4.67)
Aggressive behaviors	9.97 (6.34)
Internalizing problems	25.97 (10.92)
Externalizing problems	18.12 (10.33)

### Suicidal expressions

Suicidal expressions are shown in Table 3. ATTS revealed that 27.27% of participants felt that their ‘life was not worth living’. Nearly one-fifth (16.26%) of the young prisoners thought of their own death whereas 12.06% expressed the wish to die. Suicide ideation, suicide planning, and suicide attempts were reported by 6.82%, 1.75%, and 2.80% of participants respectively.

**Table 3.** Prevalence of suicidal expressions among young prisoners, Cambodia

Suicidal expression	Categories	Frequency (N)	Percentage (%)
Not worth living	Yes No	156 416	27.27 72.73
Death thought	Yes No	93 479	16.26 83.74
Death wishes	Yes No	69 503	12.06 87.94
Suicide ideation	Yes No	39 533	6.82 93.18
Suicide plan	Yes No	10 562	1.75 98.25
Suicide attempt	Yes No	16 556	2.80 97.20

### Factors associated with mental health problems

The following variables were inversely associated with the total YSR score both in the crude and adjusted analyses (Table 4): age, level of education and the duration of incarceration. Prisoners aged between 20 and 24 reported significantly lower mental health problems than the younger prisoners (β = −0.09; 95% CI = −0.17, −0.01). Those categorised as high education (secondary school & university graduation) revealed fewer mental health problems (β = −1.15; 95% CI = −0.23, −0.07) compared to those with a less education (primary or no school). The prisoners incarcerated for more than one year reported noticeably fewer mental health problems (β = −0.09, 95% CI = −0.18, −0.01) compared to those who had been imprisoned for less than one year.

**Table 4.** Factors associated with YSR and suicidal expressions among young prisoners, Cambodia

Description	Total YSR scores				Death thought		Suicide Ideation	Suicide Attempt		
	Crude		Adjusted		Crude		Crude		Crude	
	Coef.	95% CI	Coef.	95% CI	OR	95% CI	OR	95% CI	OR	95% CI
**Age**										
*15 to 19*	Ref.				Ref.		Ref.		Ref.	
*20 to 24*	-0.12	**-0.20, -0.04**	-0.09	**-0.17, -0.01**	1.60	0.20, 2.60	1.03	0.53, 2.01	1.07	0.40, 3.00
**Marital status**										
*Single*	Ref.				Ref.		Ref.		Ref.	
*Married*	0.01	-0.13, 0.14			1.31	0.67, 2.57	0.91	0.31, 2.67	1	
*Separated/Divorced*	0.04	-0.16, 0.24			1.64	0.64, 4.21	0.53	0.07, 4.03	1.25	0.16, 9.85
**Education**										
*Low education*	Ref.				Ref.		Ref.		Ref.	
*Higher education*	-1.16	**-0.24, -0.08**	-0.15	**-0.23, -0.07**	0.88	0.56, 1.37	0.50	**0.26, 0.98**	0.49	0.18, 1.38
**Employment status**										
*Yes*	Ref.				Ref.		Ref.		Ref.	
*No*	-0.01	-0.10, 0.09			0.87	0.52, 1.46	0.99	0.47, 2.08	0.40	0.09, 1.79
**Religion**										
*Buddhist*	Ref.				Ref.		Ref.		Ref.	
*Other*	0.07	-0.09, 0.22			1.68	0.78, 3.55	1.97	0.73, 5.34	1.84	0.40, 8.39
**Types of crimes**										
*Acquisitive crime*	Ref.				Ref.		Ref.		Ref.	
*Sexual abuse*	-0.13	-0.30, 0.30				0.71, 4.34		0.98, 9.03		0.12, 15.16
*Drug related*	-0.09	-0.19, 0.01			0.75	0.83, 2.85	2.97	0.42, 2.53	1.34	0.42, 8.96
*Violent crime*	-0.01	-0.16, 0.14						0.30, 3.76		0.26, 13.61
*Others*	-0.20	-0.41, 0.01			1.55 0.98 1.22	0.39, 2.46 0.37, 4.01	1.04 1.06 1.29	0.25, 6.59	1.93 1.97 2.27	0.20, 25.96
**Time spent in prison so far** *Less than one year* *More than one year*	Ref. -0.11	**-0.20, -0.02**	-0.09	**-0.18, -0.01**	Ref. 1.23	0.77, 1.97	Ref. 0.88	0.43, 1.82	Ref. 0.75	0.24, 2.36
Time continued to stay in prison										
*Less than one year*	Ref.				Ref.		Ref.		Ref.	
*More than one years*	-0.11	-0.20, -0.19			1.07	0.68, 1.68	1.67	0.87, 3.22	1.55	0.57, 4.19
Been in the prison before										
*No*	Ref.				Ref.		Ref.		Ref.	
*Yes*	-0.13	-0.28, 0.02			0.61	0.23, 1.58	0.60	0.14, 2.58	1	
Drug use										
*No*	Ref.				Ref.		Ref.		Ref.	
*Yes*	0.04	-0.06, 0.14			2.08	**1.12, 3.87**	1.20	0.54, 2.68	1.34	0.37, 4.76
Alcohol consumption										
*No*	Ref.				Ref.		Ref.		Ref.	
*Yes*	0.02	-0.07, 0.11			0.73	0.46, 1.16	1.38	0.66, 2.90	1.41	0.45, 4.44

### Factors associated with suicidal expressions

The risk factors associated with suicidal expressions are presented in Table 4. Drug use and education were found to be significantly associated with different suicidal expressions. Prisoners who used drugs prior to imprisonment thought about death significantly more than their counterparts did (OR = 2.08; 95% CI = 1.12, 3.87) and suicide ideation was significantly lower among prisoners with higher education (OR = 0.50, 95% CI = 0.26, 0.98). No significant associations were found between the different variables and suicide attempts.

## Discussion

This first ever study conducted on mental health problems and suicidal expressions among young male prisoners in Cambodia highlights the public health significance of this topic. Age, educational background, and duration of incarceration were significantly associated with current mental health problems. Suicidal expressions such as death thoughts and suicide ideations were significantly correlated with previous drug use and low levels of education respectively.

### Mental health problems

Overall, there is a lack of comparable studies regarding mental health among youth prisoners in LMICs. Our study showed higher YSR scores than a previous study conducted among schoolchildren in Cambodia [38]. This pattern of higher YSR among prisoners is common in the literature [42–45]. A systematic review among juvenile detention and correction facilities reported that overall mental health problems were 10 times higher than in the general adolescent population [42]. The internalising problems scores and the sub-domains such as anxiety/depression, withdrawal/depression and somatic complaints were similar to a study among Jordanian detained youth, which reported clinically significant YSR scores [43]. Both the above studies reported higher externalising problems than the general population. This is similar to the Cambodian context as the externalising problems among young prisoners in Cambodia were higher than among young people in the schools. A study from Malaysia reported 59% of juvenile offenders having conduct disorders and substance use disorders (SUD) as the most common co-morbidities [18]. In similar lines, 76% of the young prisoners in our study reported drug use prior to incarceration and the YSR mean scores related to rule-breaking and aggressive behaviours were higher than the other YSR syndromes with the exception of anxiety/depression.

The extent of mental health problems in our study is comparable in certain aspects to those reported in Sweden and the USA among young prisoners [14,46]. The young prisoners in Cambodia reported more mental health problems (YSR – 67.34) than their counterparts in the USA (YSR – 51.1). The young prisoners from the USA also reported significantly more rule-breaking behaviour than the normative population [46] which is similar to the Cambodian context as the rule-breaking behaviour among young prisoners was higher than among the schoolchildren [38]. The Swedish study that looked at the relationship between childhood-onset conduct disorder and mental health problems among young adults reported no somatisation disorder unlike our study. The Swedish study used however a different instrument whereas the study from the US, which used YSR, also reported low somatic complaints scores [46]. This could also be interpreted in the backdrop of varied perceptions of stress and mental health in different settings. It is known that somatisation can be understood as a way of expressing distress and trauma in LMICs; as a result, somatic complaints are predominantly reported [47]. In addition to this, it is also possible that there are cultural differences in reporting mental health problems due to prevailing attitudes and stigma towards mental illness [48].

We found that the mental health problems were strongly associated with younger age, low level of education and shorter-stay in the prisons. Young prisoners in Portugal reported fewer mental disorders if they had higher level of education and if their prison stay exceeded six months, which is similar to our study. However, in this study, age was not associated with mental health problems unlike ours [15]. In contrast to the Portuguese study, Kolivoski et al [49] who examined the relationship between age and misconduct behaviour among the juveniles in the US reported that those with younger age had more behavioural problems than the older, which support our findings.

### Suicidal expressions

This study reported a lower prevalence across all types of suicidal expressions except suicide attempts compared to the school study in Cambodia; for example, 16.26% of the young prisoners reported death thoughts as opposed to 25.30% by the school students [38] and 2.80% suicide attempts compared to 0.6% in the school study [38]. The higher rates of suicide attempts could be understood in the background of juvenile delinquents having a combination of externalising and internalising problems, which make them highly vulnerable [50]. The young prisoners from a Pakistani study [25] reported higher prevalence of suicidal thoughts (22%) than in our study (7%), thought the age groups were slightly different. Another study from Australia among young offenders revealed that 16% and 10% of their participants reported suicide thoughts and suicide attempts respectively [24], higher than in our study. These differences could be explained due to potential underreporting as the young prisoners may hesitate to disclose their suicidal expressions for fear of being confined in a separate room under constant vigilance and separated from others as per the prison regulation to prevent suicides. It is also possible that religion and the related belief system about after life in Cambodia, being predominantly Buddhist, may contribute to lower reporting of suicidal expressions [51], an issue that needs further exploration.

This study found that death thoughts and suicide ideation significantly correlated with a history of drug use and low level of education, respectively. The studies from Australia and Pakistan found that suicidal expressions among young offenders were neither associated with pre-incarceration drug use nor with the level of education [24,25]. The difference in the associations between history of drug use and suicidal expressions could be due to distinct levels of perceptions of stress and coping mechanisms while in the prisons, which may vary in different cultural contexts [52]. Though among imprisoned women, Zhong et al [53] found an association between low educational level, history of drug use and increased suicide risks in China which underscores the need for understanding the complexity of age, drug use and suicide from a gender perspective. Further research, particularly qualitative studies, are warranted to elucidate why drugs and educational level were associated with suicidal expressions among young prisoners in Cambodia.

## Limitations

To the best of our knowledge, there has not been any research on the mental health of young prisoners in Cambodia and therefore there is no opportunity to make comparisons. The findings from the three prisons located in the central, north, and south of the country, may not reflect the situations in other prisons and therefore cannot be generalised to the entire nation. Lack of opportunity to examine the data from a gender perspective was a major limitation, as we had to exclude the female prisoners due to the negligible number. The mental health questionnaires (YSR and ATTS) were administered by face-to-face interviews in the context of poor literacy level among the participants, which could have introduced respondent bias. As previously mentioned, it is possible that the young prisoners might have under-reported suicidal expressions because of the concerns of being penalised.

## Conclusion

The situation of mental health problems and suicidal expressions among young prisoners in Cambodia is a public health concern demanding comprehensive mental health services that are integrated within the current prison health system. The age, educational status, the duration of incarceration and a previous history of drug use need to be taken into account while instituting routine mental health assessments and while implementing contextualised interventions.

## Paper context

Young prisoners are most vulnerable to mental health problems and suicidal expressions. Yet, no studies have been conducted in Cambodia. The situation of mental health problems and suicidal expressions among young prisoners indicate the need for comprehensive and well-planned mental health services that are integrated within the prison health system in Cambodia.
